# Simulating the Fate of Dimethyl Sulfide (DMS) in the Atmosphere: A Review of Emission and Chemical Parameterizations

**DOI:** 10.3390/atmos16030350

**Published:** 2025-03-20

**Authors:** Ernesto Pino-Cortés, Mariela Martínez, Katherine Gómez, Fernando González Taboada, Joshua S. Fu, Golam Sarwar, Rafael P. Fernandez, Sankirna D. Joge, Anoop S. Mahajan, Juan Höfer

**Affiliations:** 1 Escuela de Ingeniería Química, Pontificia Universidad Católica de Valparaíso, Valparaíso 2362854, Chile; 2 Escuela de Ciencias del Mar, Pontificia Universidad Católica de Valparaíso, Valparaíso 2373223, Chile; 3 Centro Oceanográfico de Gijón, Instituto Español de Oceanografía-CSIC, 33212 Asturias, Spain; 4 Department of Civil and Environmental Engineering, University of Tennessee, Knoxville, TN 37796, USA; 5 Center for Environmental Measurement & Modeling, U.S. Environmental Protection Agency, Durham, NC 27711, USA; 6 Institute for Interdisciplinary Science, National Scientific and Technical Research Council (ICB-CONICET), Mendoza 5501, Argentina; 7 Centre for Climate Change Research, Indian Institute of Tropical Meteorology, Pune 411008, India; 8 Department of Atmospheric and Space Sciences, Savitribai Phule Pune University, Pune 411007, India

**Keywords:** DMS, ocean flux, regional simulation, global modeling, air quality modeling

## Abstract

Numerical simulation studies of the dispersion of dimethyl sulfide (DMS) in the air have increased over the last two decades in parallel with the interest in understanding its role as a precursor of non-sea salt aerosols in the lower to middle levels of the troposphere. Here, we review recent numerical modeling studies that have included DMS emissions, their atmospheric oxidation mechanism, and their subsequent impacts on air quality at regional and global scales. In addition, we discuss the available methods for estimating sea–air DMS fluxes, including parameterizations and climatological datasets, as well as their integration into air quality models. At the regional level, modeling studies focus on the Northern Hemisphere, presenting a large gap in Antarctica, Africa, and the Atlantic coast of South America, whereas at the global scale, modeling studies tend to focus more on polar regions, especially the Arctic. Future studies must consider updated climatologies and parameterizations for more realistic results and the reduction in biases in numerical simulations analysis.

## 1. Introduction

Oceans are the largest natural source of sulfate aerosols [1], accounting for an annual input of 27.1 Tg of sulfur to the atmosphere [2]. They are only second in importance to marine aerosol emissions of sea salt particles released through sea spray [3]. Most oceanic sulfate aerosols are produced by the oxidation of the primarily emitted biogenic gaseous compound, dimethyl sulfide ($\text{DMS-}{\text{CH}}_{3}{\text{SCH}}_{3}$), which is released as a by-product of phytoplankton activity and decay [4]. DMS acts as a precursor of sulfate and sulfonate obtained after atmospheric degradation by several chemical reactions.

DMS is considered the main precursor of non-sea salt sulfate aerosols, which contribute a net climate forcing about −1.7 W m^−2^ to Earth’s global climate system [5]. These non-sea salt sulfate aerosols are involved in both direct negative radiative forcing [5,6] and cloud condensation nuclei (CCN) in the atmosphere [7]. AerChemMIP (Aerosols and Chemistry Model Intercomparison Project) simulation experiments conducted in the context of the last IPCC (Intergovernmental Panel on Climate Change) assessment report (AR6) identified DMS as a potential positive biogeochemical feedback mechanism on the climate [8]. In addition, DMS has been associated with urban aerosols in coastal cities such as Los Angeles, USA [9], Shanghai, China [10], and Quintero, Chile [11]. Very recently, the coupling of DMS and methanethiol emissions has recently been suggested to enhance as much as 30–70% of the sulfate aerosol burden over the southern ocean, highlighting the importance of improving the regional distribution and chemical degradation pathways of sulfur oceanic emissions [12].

The study of Barnes et al. (2006) provided a comprehensive overview of the chemical reactions involved in the oxidation of DMS in the troposphere [13]. DMS is a short-lived pollutant with a lifetime of 1–2 days due to its high reactivity in the air [14]. Three main gas phase (3GP) DMS oxidation pathways have been identified:

(1) $${\text{DMS}}_{\left(\text{g}\right)}+{\text{OH}}_{\left(\text{g}\right)}\overset{\text{abstraction}}{\to }{\text{SO}}_{2(\text{g})}+{\text{CH}}_{3}{\text{O}}_{2}+{\text{CH}}_{2}\text{O}$$

(2) $${\text{DMS}}_{\left(\text{g}\right)}+{\text{OH}}_{\left(\text{g}\right)}\overset{\text{addition}}{\to }0.75{\text{SO}}_{2(\text{g})}+0.25{\text{MSA}}_{\left(\text{g}\right)}$$

(3) $${\text{DMS}}_{\left(\text{g}\right)}+{\text{NO}}_{3(\text{g})}\to {\text{SO}}_{2(\text{g})}+{\text{HNO}}_{3}+{\text{CH}}_{3}{\text{O}}_{2}+{\text{CH}}_{2}\text{O}$$

where DMS = dimethyl sulfide, OH = hydroxyl radical, ${\text{SO}}_{2}$ = sulfur dioxide, ${\text{CH}}_{3}{\text{O}}_{2}$ = methyl peroxy radical, MSA = methanesulfonic acid, ${\text{NO}}_{3}$ = nitrate radical, and ${\text{HNO}}_{3}$ is nitric acid.

During the daytime, DMS primarily reacts with OH (through abstraction and addition pathways), whereas it reacts with ${\text{NO}}_{3}$ radicals at night [15]. It is relevant to note that abundant ${{\text{NO}}_{3}}^{-}$ is observed in polluted areas when NOx emissions from anthropogenic sources are present (e.g., power energy stations and transportation).

In addition to the 3GP reactions, direct DMS oxidation reactions with ${\text{O}}_{3}$, halogen radicals Cl, Br, and halogen compounds like BrO, ClO, and IO have also been analyzed [16,17]. Indeed, over the past two decades, many air quality modeling studies have focused on DMS, along with a growing interest in the tropospheric chemistry of very short-lived (VSL) species. The repeated conclusions and suggestions of those studies were the need to include two main research lines to be addressed: DMS chemistry in the atmosphere and the development of accurate emission inventories. Note that despite the fact that direct oxidation pathways of DMS driven by halogen chemistry exist, the mayor influence of VSLs on the degradation of voltaic organic compounds (including DMS) is the indirect halogen-driven enhancement of OH [18].

This review aims to identify studies and model findings related to DMS emissions and the effects in the air of using numerical simulation models. We also encourage the establishment of a baseline and future perspectives on this topic.

## 2. Estimation of the DMS Emission Inventories and Climatologies

The annual emissions of marine DMS have been estimated to be approximately half of global sulfur emissions [19]. DMS production is biogenic; it is formed from the chloroplasts inside phytoplankton from its precursor dimethysulfoniopropionate (DMSP), which is a molecule used to regulate cell buoyancy and prevent ice formation [20–22]. Once in water, DMS can follow several pathways: undergoes photolysis to generate dimethyl sulfoxide (DMSO)—a substrate for bacteria consumption getting reduced to elemental sulfur which is the most frequent pathway—or exchange to the atmosphere, creating an sea–air flux (F).

One of the most widely used methodologies to obtain the sea–air flux of DMS considers sea–air exchange [23] as follows:

(4) $$F=k_{T}\cdot \left(C_{W}-C_{g}\cdot H\right)$$

where $C_{W}$ and $C_{g}$ correspond to the concentration of DMS in water and air, respectively, which are related by the Henry’s Law constant (H).

The factor $k_{T}$ is the gas transfer coefficient and depends on the total resistance to gas transfer on both sides of the air $\left(k_{a}\right)$/sea $\left(k_{w}\right)$ interface, the temperature of the air $\left(T_{a}\right)$, the Henry constant (H), and the universal gas constant (R), as shown in Equation (5):

(5) $$k_{T}={\left(\frac{1}{k_{w}}-\frac{R\cdot H\cdot T_{a}}{k_{a}}\right)}^{-1}$$

For DMS, H (expressed in atm L mol^−1^) is related to seawater temperature (T expressed in kelvins) as follows [24]:

(6) $$\text{ln}(H)=-\frac{3457}{T}+12.64$$

The factor $k_{a}$ is usually avoided when only water side resistance is assumed. This is due to DMS concentration in the waterside usually being higher than in the airside. However, this parameter is significant at cold temperatures and high wind speeds [25]. This factor can be obtained using the following equation:

(7) $$k_{a}=659\cdot U_{10}\cdot {\left(\frac{62}{18}\right)}^{0.5}$$

The gas transfer efficiency mainly varies with the water resistance coefficient $k_{w}$ (in cm·h^−1^), which essentially depends on the wind speed. Table 1 presents the most commonly used parameterizations of $k_{w}$ as a function of wind speed at 10 m $\left(U_{10}\right)$.

The LM86 parameterization was derived from experiments in wind tanks, whereas the N00 and H06 equations were obtained through measurement techniques in the open ocean. The rest of the parameterizations (W92, W99, M09, and W14) applied the eddy covariance method to their estimation. Further details and differences can be found in Joge et al. (2024) [33]. As shown in Table 1, all $k_{w}$ parameterizations consider the unitless normalized Schmidt number $\left(Sc_{N}\right)$, which depends on the sea surface temperature and the substance of analysis [34].

Alternatively, Jones et al. (2004) proposed the following equation to estimate the DMS flux from the ocean using the parameterization W92 [35]:

(8) $$F=\frac{8}{9}\cdot 10^{-13}\cdot k_{w}\cdot C_{W}$$

The development of the term $C_{W}$ and extrapolation to obtain a global database of DMS emissions has been a topic of interest since the 1980s. One of the first climatologies was conducted by Bates et al. (1987) using more than 1000 samples in the Pacific Ocean [36]. This study served as the basis for others [37,38]. The last reference added concentrations from the Southern Ocean but yielded a negligible impact. Subsequently, several studies were conducted with the same goal. The study from Belviso et al. (2004) compared seven sources of climatology of data showing differences for specific zones of analysis [39]. The dataset reported by Kettle and Andreae (2000), an update of the climatology data shown in Kettle et al. (1999) [40], was the reference for the comparison owing to more than 15,000 measurements of DMS seawater concentrations [41].

Other methodologies have also been applied to estimate the DMS flux from the ocean. Considering unidirectional flux, the amount of DMS emitted could be estimated using atmospheric observations of this substance based on the mass balance in a well-mixed boundary layer of air and its photochemistry. The studies of Chen et al. (1999) and Davies et al. (1999) [42,43] proposed the following equation:

(9) $$\frac{d[DMS]}{dt}=\frac{F_{DMS}}{EMD}-\left(k_{OH}[OH]+k_{{NO}_{3}}\left[{NO}_{3}\right]\right)[DMS]$$

EMD is the DMS equivalent mixing depth representing the height of a column with all DMS masses in the air. Equation (9) assumes that DMS reacts only with OH and ${\text{NO}}_{3}$, and if the photochemical reactions are well described, the flux can be estimated. For instance, Shon et al. 2005 [44] used the mass balance photochemical approach to analyze DMS emissions around Jeju Island (33.17° N, 126.10° E), South Korea, during Asia Dust Storm (4.4 μmole m^−2^ day^−1^) and Non-Asia Dust Storm (2.4 μmole m^−2^ day^−1^).

In recent years, some studies have been conducted to obtain new inventories, particularly in regions with unknown data or high uncertainties. The study of Chen et al. (2018) [17] reported 22 Tg S yr^−1^ globally for 2017 using the sea surface DMS concentration obtained from Lana et al. (2011) [19] and 18 Tg S yr^−1^ using the data from Kettle et al. (1999) [40]. Therefore, Ogunro et al. (2018) [45] created an International Ocean Model Benchmarking (IOMB) package using the Python (version 3) programming language, considering measurements for the period between 1978 and 2008. The work was developed using the parameterization N00 [29] and compared with the dataset in Lana et al. (2011) [19]. Recently, marine DMS emissions from East Asian seas were estimated during the period 2014–2016, with an average flux of 0.73 μmole m^−2^ day^−1^, a maximum of 3.4 μmole m^−2^ day^−1^ (December 2014), and a minimum of 0.12 μmole m^−2^ day^−1^ (March 2014) [6].

From the first interpolation-based climatology data shown in Kettle et al. (2000) [41] to the latest developed by Hulswar et al. (2022) [2], DMS observations have drastically increased. Instead of using direct DMS observation, most recent studies have developed climatologies using satellite-based proxy parameters [46,47]. There is a large degree of uncertainty between these three DMS climatologies. The area-weighted global annual DMS concentration estimated by Hulswar et al. (2022) [2] was 2.28 nM, while Galí et al. (2018) [47] and Wang, et al. (2020) [46] estimated 1.69 nM and 1.75 nM, respectively. In addition, region-wise, there is large uncertainty regarding the seawater DMS estimates [33]. There are regions where the total uncertainty regarding the DMS flux is due to DMS seawater concentrations which are larger than the other regions where the uncertainty is due to the choice of the flux parameterization method [33]. Also, in the CMIP6 (Coupled Model Intercomparison Project) models (NorESM2-LM, CNRM-ESM2–1, UKESM1–0-LL, and MIROC-ES2L), there is a large degree of uncertainty between the DMS seawater estimates and the sea–air DMS flux for the historical as well as future scenarios [48].

An alternative option for the inclusion of ocean DMS emissions for air quality models is the Copernicus Atmosphere Monitoring Service (CAMS) database. The CAMS has been developed with freely and available emission inventory for DMS and halocarbons, within the CAMS-GLOB-OCE (Global oceanic emissions of DMS, OCS, and halogens) dataset. The flux for the DMS emissions was obtained using the climatology data reported in Lana et al. (2011) [19]. It is available in the Emissions of Atmospheric Compounds and Compilation of Ancillary Data (ECCAD) website (http://eccad.aeris-data.fr/#DatasetPlace:CAMS-GLOB-OCE$DOI accessed on 28 February 2023).

## 3. Modeling of DMS Chemistry in the Atmosphere

### 3.1. Regional Models

In the last 25 years, regional modeling studies considering the DMS flux and its chemistry have been limited to the use of Weather Research and Forecasting coupled with Chemistry (WRF-Chem) [49], Community Multiscale Air Quality (CMAQ) [50], the Environment—High Resolution Limited Area Model (Enviro-HIRLAM) [51], the Global Environmental Multiscale—Modelling Air quality and Chemistry (GEM-MACH) [52], and European Monitoring and Evaluation Programme for United Kingdom (EMEP4UK) models. All models require emission estimates data for further processing. DMS emission processing methodologies applied in regional air quality simulation are described below:

- Offline approach: This method estimates the DMS emission outside the air quality model, which is a crucial stage before modeling. The generated file must contain the geographic information of the analysis domain and temporal variation during the period of study. As a benefit, the obtained emission inventory can be applied to different models. One way to apply this method is to estimate DMS emissions from monitoring campaign studies, providing an extrapolation of those values. Considering this approach, the study of Kazil et al. (2011) [53] used the VOCALS-Rex (VAMOS Ocean-Cloud-Atmosphere-Land Study Regional Experiment) campaign, while Mueller et al. (2011) [54] applied constant values from Kloster et al. (2006) [55]. We highlight that the GOCART (Goddard Chemistry Aerosol Radiation and Transport) dataset was the preferred input in several studies [56–60].
- Inline approach: This method calculates DMS emissions within the atmospheric models during the simulation. This alternative requires the spatial distribution of the DMS seawater concentration, which is combined with meteorological variables and parameterizations to obtain the factor $k_{w}$. In this approach, constant input values have been considered [61–63], giving variability to the inline flux emission only according to the selected parameterization as functions of the meteorological variables present in the zone. However, a method that is closer to reality is the application of time–space-varying DMS seawater concentration maps applied in several studies [9,10,16,64–71].

Table 2 presents the published studies including DMS emissions and their chemistry in regional simulation studies.

**Figure 1.**
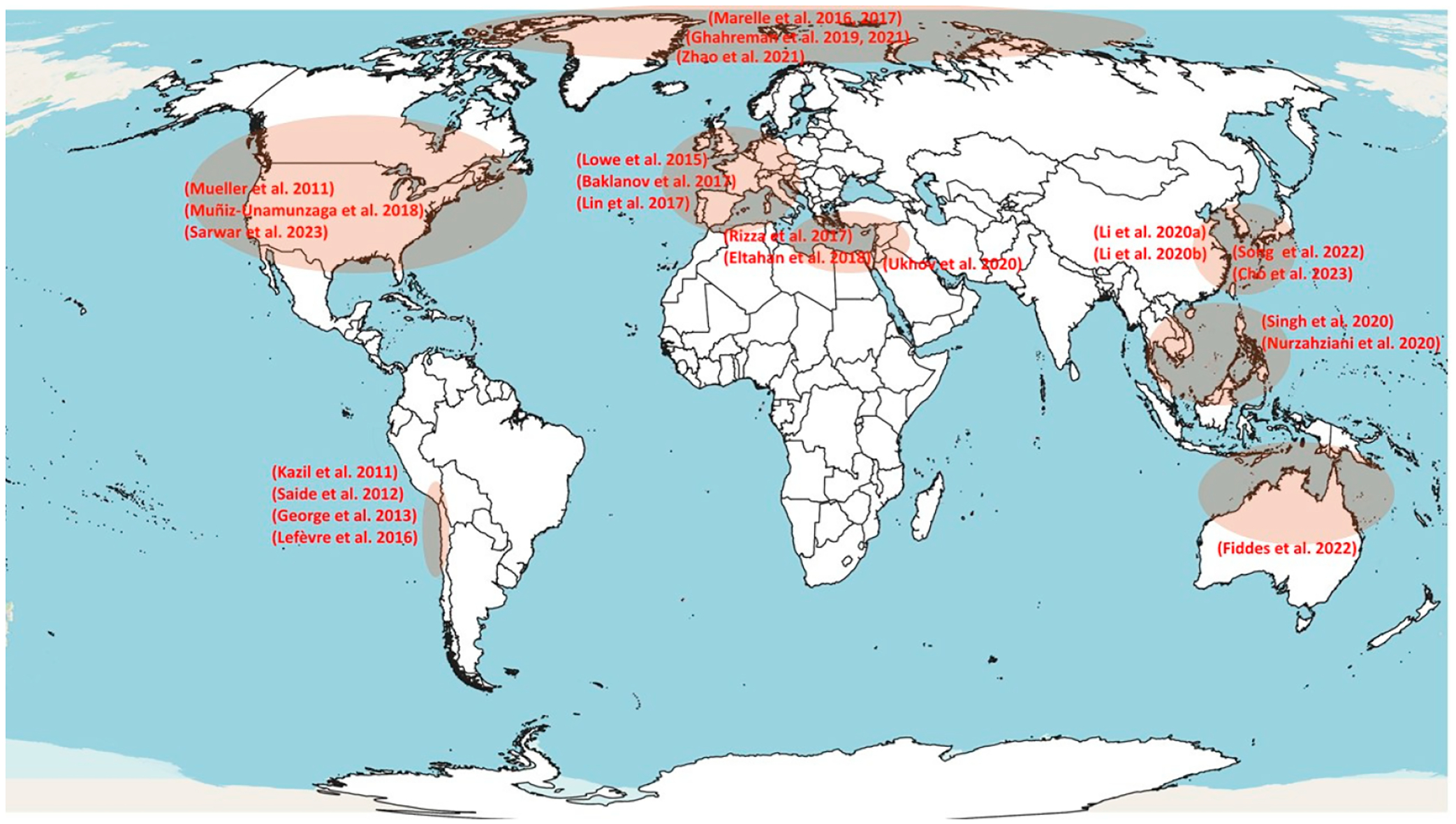
Regions analyzed in numerical simulation studies including DMS emissions. Muñiz-Unamunzaga et al. (2018) [9], Li et al. (2020a) [10], Zhao et al. (2021) [16], Kazil et al. (2011) [53], Mueller et al. (2011) [54], Rizza et al. (2017) [56], Eltahan et al. (2018) [57], Singh et al. (2020) [58], Ukhov et al. (2020) [59], Nurzahziani et a. (2020) [60], Saide et al. (2012) [61] George et al. (2013) [62], Lowe et al. (2015) [63], Li et al. (2020b) [64], Song et al. (2022) [65], Cho et al. (2023) [66], Sarwar et al. (2023) [67], Fiddes et al. (2022) [68], Marelle et al. (2017) [69], Marelle et al. (2016) [70], Lefèvre et al. (2016) [71], Baklanov et al. (2017) [74], Lin et al. (2017) [76], Ghahreman et al. (2019) [77], Ghahreman et al. (2021) [78].

Figure 1 shows the regions analyzed in each of the studies using DMS emissions, and the chemistry models are described in Table 1. The studies were mostly concentrated on the Northern Hemisphere, specifically on the coasts of the United States, the Arctic, Western Europe, the Mediterranean Sea, China, South Korea, and Southeast Asia. In the Southern Hemisphere, studies only have investigated regions in Australia and the coasts of Peru and Chile.

In the following section, an overview of the main studies using the CMAQ and WRF-Chem models is presented considering the timescale of updates to the models.

#### 3.1.1. CMAQ

During the last 20 years, the CMAQ model has been developed and updated, including its complex gas phase photochemical mechanism. The study by Mueller et al. (2011) [54] was the first simulation to include DMS using the CMAQ model (version 4.6). As a result, DMS emissions and chemistry showed that the aerosol sulfate increased by nearly 2 μg·m^3^ in ocean and 0.1–0.2 μg·m^3^ in inland areas of North America in 2002. This study highlighted the need for the inclusion of natural emissions, especially from the ocean, when evaluating coastal areas. However, years later, no study included DMS emissions in CMAQ simulations until the research study of by Muñiz-Unamunzaga et al. (2018) [9]. This was the first study in an urban coastal zone in the USA with halogens and DMS emissions included using the CMAQ model (version 5.1) at a city scale (4 km of resolution). The authors added the DMS and halogen chemistry to the open-source code and observed an increase of 10% in secondary organic aerosol mean concentration due to aerosol acidity and sulfate aerosol formation. Otherwise, the ozone and nitrogen dioxide $\left({\text{NO}}_{2}\right)$ concentrations diminished by up to 5 ppbv and 2.5 ppbv, respectively, when marine emissions were considered.

Years later, Li et al. (2020b) used the CMAQ model (version 5.2) but analyzed the coast of Shanghai, China [64]. In this case, the inclusion of marine emissions of DMS and halogens increased the formation of sulfate aerosols on the coast by 4% and at sea by 9%. We note that the authors used a Chinese database for DMS seawater concentration (compiled from cruise survey experiments from 2009 to 2017) instead of climatology data from Lana et al. (2011) [19]. The main reason was the more realistic and confident data in the region of analysis from the local database than from the global dataset. One of the main conclusions was that marine emissions should be included in future air quality modeling studies in coastal areas to improve the accuracy of the predictions when using models like CMAQ.

Recently, Zhao et al. (2021) [16] used the Integrated Reaction Rate (IRR) option in the CMAQ model (version 5.3) to estimate the relative contribution of each of the seven reactions involving DMS and oxidants (OH, ${\text{NO}}_{3}$, Cl, ClO, IO, and BrO) to the total DMS oxidation rate. Their results indicate the importance of including both halogen species (Cl, Br, and I) and DMS emissions in their reaction pathways. These coupled chemistry modeling approaches have usually been avoided in past studies on air quality modeling. The results suggested that 63.5% of DMS was oxidized by OH (33.0% via the abstraction channel and 30.5% via the addition channel). Also, the oxidation of DMS by ${\text{NO}}_{3}$ accounted for 11.8%. Therefore, BrO, Cl, IO, and ClO oxidation pathways contributed 16.0%, 8.2%, 0.4%, and 0.1%, respectively, to the total DMS oxidation.

The CMAQ model was updated to version 5.3.1 [79] to include DMS chemistry and other changes. Recently, Song et al. (2022) used the CMAQ model (version 5.3.2) to evaluate the effect of ships and DMS emissions in South Korea and surrounding sea areas during 2017–2018 [65]. The authors estimated the DMS concentration in surface seawater using an empirical algorithm combining the chlorophyll a (Chl-a) concentrations (from http://modis-atmos.gsfc.nasa.gov/ accessed on 28 September 2024) and the mixing layer depth. In that study, the IRR option showed that 7–9% of DMS was oxidized by OH (62.9% for reaction 1 and 37.1% for reaction 2) and 91–93% by ${\text{NO}}_{3}$. Meanwhile, in open oceans, the last percentage decreased to 15–29% owing to low NOx levels. In a different way, DMS contribution to the total PM_2.5_ was approximately 1%, showing more influence in the coastal area than the open ocean. As an interesting conclusion, the authors reported substantial differences between the modeled results and observational data and suggested that the omission of halogen chemistry during simulations and uncertainties in DMS flux contributed such differences, which should be studied in future modeling research in that region.

The impact of oceanic DMS emissions on sulfate concentrations over the continental U.S. for recent atmospheric conditions using the CMAQ model (version 5.4) was analyzed by Sarwar et al. (2023) [67]. That study showed that a higher impact was present in near coastal areas than over inland areas. The largest impacts over land were in the coastal areas of Washington, Oregon, California, southern Texas, and Florida, where the typical increase in sulfate concentrations was more than 30–40%. In addition, the authors showed that DMS emissions increased the annual mean sulfate concentration by 0.055 μg/m^3^ over the land area of the modeling domain. The main conclusion was that future studies in coastal zones should consider marine emissions for air quality simulations.

#### 3.1.2. WRF-Chem

Kazil et al. (2011) [53] were the first to use DMS chemistry in the air using the WRF-Chem model. In this case, the authors added reactions 1 and 3 with the temperature-dependent rate coefficients derived from Hynes et al. (1986) [80] and Atkinson et al. (1992) [81], respectively. The DMS flux from the ocean was set from the VAMOS Ocean-Cloud-Atmosphere-Land Study Regional Experiment (VOCALS-REx) conducted on October and November of 2008 considering two scenarios: 4.8 μmol m^−2^ d ^−1^ and 2.4 μmol m^−2^ d ^−1^. In addition, 60 and 25 ppt were assumed as the DMS concentrations in the boundary layer. These assumptions were distinguished in the uncertainties of the simulation (48 h starting on 27 October 2008, domain center at 80° W, 20° S). However, the results from the first scenario were consistent with the surface measurements at the shipboard. The second test showed good agreement with aircraft data. The main reason for this was the nighttime oxidation of DMS with ${\text{NO}}_{3}$.

Saide et al. (2012) attempted to include DMS emissions from the marine evaluation of the WRF-Chem model against the VOCAL-Rex campaign [61]. As previously analyzed, the authors assumed a constant DMS seawater concentration (2.8 nM·L^−1^) but employed the sea–air exchange reported by Liss and Merlivat (1986) [26]. Among other results, that study overestimated the DMS emissions due to overestimating the modeled DMS in the ocean by the atmospheric transfer velocity, generating high biases in the modeled marine boundary layer and increasing cloud-driven ${\text{SO}}_{2}$-to-sulfate conversion.

The constant seawater DMS concentration was repeated in the work of George et al. (2013) [62]; however, these authors used the parameterization of Nightingale et al. (2000) [29] to estimate the DMS flux. The chemical reactions were the same as those used in previous studies using the WRF-Chem model. The focus of the study was the evaluation of DMS emissions during the VOCALS-REx campaign and simulated the period from 15 October to 16 November 2008 in a nested domain spanning 3–40° S and 65–95° W. As a result, an overestimation of the DMS flux was obtained, but it had an impact on the albedo. In addition, the simulation results suggested that DMS influenced the aerosol number and size distribution. This research and the study of Saide et al. (2012) [61] could be considered pioneers with respect to investigating the impact of DMS in the southern coast of the Pacific Ocean.

Later, Lowe et al. (2015) [63] reduced the DMS seawater concentration to 2 nM·L^−1^ and performed simulation over the United Kingdom in July 2010. The authors assumed this value according to the lowest value based on the climatology of Kettle et al. (1999) [40]. As a result, the nighttime ${\text{NO}}_{3}$ oxidation of DMS was more relevant than the daytime OH reaction. This particular finding probed the biases found before the DMS chemistry and the required updates for future studies.

The inclusion of DMS emissions and chemistry in WRF-Chem model studies continued in 2017. The study of Marelle et al. (2017) [69] adopted the inline approach in the model to calculate DMS emissions using the climatology by Lana et al. (2011) [19] and the parameterization of Nightingale et al. (2000) [29]. The authors focused on the Arctic region analyzing a hemispheric domain, showing a high source contribution (90–100%) of DMS to surface ${\text{SO}}_{2}$ in this region. In addition, they determined several improvements in the performance of the model for ${\text{O}}_{3}$ and surface sulfate but noted that the simple gas phase chemistry had an impact on overestimation in some cases.

In contrast to the studies mentioned previously, Rizza et al. (2017) [56] considered the GOCART dataset as the prescribed input (offline approach) to obtain the DMS flux and subsequently assumed the climatology reported by Kettle et al. (1999) [40] to evaluate the same model in the central Mediterranean region during May 2014. The authors mainly analyzed dust and particulate matter, revealing an overestimation of PM_2.5_ when the dust module was considered, but no discussion was reported on the effect of marine emissions.

The offline method, using the same GOCART database as the input to estimate DMS emissions in the WRF-Chem model, was also considered in four different studies in 2018–2020. Eltahan et al. (2018) [57] centered on the simulation of two severe dust storms in Egypt in January 2004 and March 2013. The study of Nurzahziani et al. (2020) [60] applied WRF-Chem version 3.5.1 using the DMS emissions based on the year 2006 in the GOCART database to simulate the period 30 January–30 April for the years 2014–2015 in Thailand evaluating PM_10_ concentrations. In another study, Ukhov et al. (2020) [59] evaluated version 3.7.1 over the Kingdom of Saudi Arabia during 2015–2016 studying ${\text{SO}}_{2}$ dispersion. Finally, Singh et al. (2020) [58] used version 3.8.1 of the model to simulate the period during the El Niño–Southern Oscillation in 2013 over South Asia, focusing on black carbon. Marelle et al. (2017) [69] did not analyze the effect of marine emissions, but it is remarkable to distinguish their inclusion in the simulation for better performance.

A recent study by Fiddes et al. (2022) [68] changed the paradigm of the methodology when the WRF-Chem model was applied. The authors used the climatology of Lana et al. (2011) [19] and the LM86 parameterization [26]. The simulation was conducted from October 1 to 25, 2016, and was centered in a regional domain in Queensland, Australia. It is remarkable to distinguish that the authors used the version designed with 30 DMS oxidation pathway reactions.

### 3.2. Global Models

The global modeling methodologies used to estimate the DMS flux are the same as those used in the regional modeling analysis. Most recent global studies applied the inline method.

According to the information presented in Table 3, in the last 15 years, there have been other global models such as GEOS-Chem, HadGEM, MIROC, CAM-Chem, NorESM2, CanAM, ECHAM, and STOCHEM. These global models are used in climate research studies. In this approach, the DMS seawater concentration and parameterization to estimate the emission estimates are relevant for obtaining simulated data with low error biases.

In the following section, an overview of the main studies using GEOS-Chem model is presented considering the timescale of updates to the models.

#### GEOS-Chem

The standard Goddard Earth Observing System—Chemistry (GEOS-Chem) global chemical transport model is widely applied on a global scale. Most of the studies have focused on polar regions in the northern and southern hemispheres. The first studies using the GEOs-Chem model used the 3GP reactions, and during the last 5 years, the simulations were updated with several chemical reactions with halogen compounds, showing more information about DMS conversion and its effect in the marine boundary layer of the atmosphere.

The study of Hezel et al. (2011) [83] applied the GEOS-Chem model (v8-01-03) to analyze different DMS climatology data from Simó and Dachs (2002) [82], Kettle et al. (1999) [40], Lana et al. (2011) [19], and their own construction. The last option considered the average concentrations in the Southern Ocean with unknown data. Therefore, the authors used the parameterization of Nightingale et al. (2000) [29] and the same gas transfer parameterization over sea ice as over the open ocean. In addition, they added the reaction between DMS- and BrO-producing ${\text{SO}}_{2}$ and MSA according to (Breider et al., 2010) [14]. As a result, several simulations were performed from 1985 through 2004 to evaluate DMS climatology data, fluxes from sea ice cores, and reactions with BrO. The main findings were a small contribution (11–30%) of sea ice extent to DMS emissions, and DMS emissions were responsible for 26–62% of MSA deposition at the Antarctic coast and 36–95% inland. The lifetimes of DMS and MSA varied with respect to whether their reaction with BrO was considered or not.

In the same year, Gray et al. (2011) [84] compared the results from the Pacific Atmospheric Sulfur Experiment (PASE) with GEOS-Chem simulations and The Regional chEmical trAnsport Model (REAM), which is a 1D transport model. The study focused on DMS flux and ${\text{SO}}_{2}$ deposition over the equatorial Pacific between 8 August and 6 September 2007. The DMS flux from the ocean was inferred from the monitoring campaign for the REAM simulation; however, the input data for the GEOS-Chem simulations were not available, suggesting the use of the same data for both models. Paradoxically, this study also analyzed the effect of the reaction of DMS with BrO. By adding 1 pptv of this oxidant, a reduction of 13% in DMS-to-${\text{SO}}_{2}$ conversion efficiency was obtained, supporting the results of Hezel et al. (2011) [83].

Leaitch et al. (2013) [85] used the GEOS-Chem model (v8-02-02), coupled with the TwO-Moment Aerosol Sectional (TOMAS) microphysics model, to analyze aerosol particle observations in the Arctic from March 2021 to March 2022. The DMS flux was estimated from the global dataset reported by Kettle et al. (1999) [40] using the parameterization of Liss and Merlivat (1986) [26]. The chemical pathway for DMS was based on the GOCART model with the reactions from Chin et al. (2002) [107]. The results showed overprediction of MSA concentrations compared to observed registries at the Alert station, which were argued by overestimation of the DMS emissions. Even when the authors suggested that cause, other explanations could be associated with missing reactions in the chemical mechanism that could reduce DMS in that region. In other analysis, the results showed the main contribution to nuclei cloud droplets (CCNs) from DMS during July–August in the region, which also impacted the aerosol cloud albedo.

Previously, the study of Mungall et al. (2016) [86] used measurements of atmospheric DMS and sensitivity simulations with the GEOS-Chem model to conclude that non-marine sources could contribute to additional episodes of this substance in the Arctic region, although local marine sources of DMS dominated. Although this study used flux data from Lana et al. (2011) [19] for the emission estimate, the authors improved the data with 21-day ship track measurements. Otherwise, it is remarkable that the study applied DMS oxidation only by reaction with OH and ${\text{NO}}_{3}$ according to Equations (1)–(3) following Leaitch et al. (2013) [85].

The sulfur oxidation mechanism of the GEOS-Chem model with the DMS chemistry proposed by J. B. Burkholder et al. (2015) [108] for gas and aqueous phase reactions was updated in Chen et al. (2018) [17]. This study included dimethyl sulfoxide (DMSO) and methane sulfonic acid (MSA) intermediates. The main results suggested the global oxidation of DMS in the gas phase by OH (66%), ${\text{NO}}_{3}$ (16%), and BrO (12%). The authors also evaluated the climatology data from Lana et al. (2011) [19] and Kettle et al. (1999) [40], obtaining 22 Tg S yr^−1^ and 18 Tg S yr^−1^, respectively, for 2007. This paper reported the presence (around 86%) of DMS in the tropospheric burden below 2 km and revealed that DMS oxidation by OH occurred during daytime in contrast to the nighttime, when ${\text{NO}}_{3}$ has more availability to react with it. The reaction with BrO was considerable in the Southern Ocean and Antarctica during winter.

Recently, Meng et al. (2019) [89] used the GEOS-Chem model showing the source contribution to secondary aerosol PM_2.5_ concentrations in Canada during 2013. The results indicated low apportionment of DMS to particulate matter.

## 4. Conclusions

According to this review, most of the modeling studies with the inclusion of DMS emissions and chemistry were done at a regional and global scale with diverse models. At the regional scale, the CMAQ and WRF-Chem models have been widely applied. However, there is a large gap of regional modeling studies in Antarctica, Africa, and the Atlantic coast of South America. The main reason could be associated with less available monitoring data, which are crucial in simulation studies.

In contrast to regional modeling studies, global modeling studies have centered on polar regions over the Arctic. The results obtained have been crucial for the understanding of the impact of marine emissions in pristine zones, focusing on climate variability at lower and middle troposphere levels. In the future, the results of global modeling studies should serve as a basis for initial and boundary conditions of regional modeling studies. It is remarkable to mention that most of the references analyzed did not mention the details about the initial and boundary conditions of DMS and by-products in the air when the regional scale was analyzed. This information is crucial to reduce biases and could reduce the uncertainty of their chemistries.

The baseline DMS chemistry applied in regional simulation studies considers the destruction of DMS by the action of OH and ${\text{NO}}_{3}$ radicals, which is the 3GP basic scheme of reactions. However, the addition of chemical reactions involving halogenated compounds reduces simulation errors, particularly in open sea areas [64]. From our point of view, this should be the chemical mechanism to be applied to avoid results that may lead to inaccurate conclusions. Most studies that have used the CMAQ model comply with adequately applying the chemical mechanism of DMS destruction in the air. However, when the WRF-Chem model was employed, the 3GP mechanism was applied, except in the study by Lowe et al. (2015) [63]. Thus, it is suggested that the model should be updated to integrate more advanced chemical mechanisms.

The climatology report of Lana et al. (2011) [19] is the most widely used reference study and the main input to obtain the DMS flux from the seawater in studies published in the recent years. However, future studies must consider updated DMS seawater concentrations. Monthly data are usually applied in both regional and global modeling studies. As a recommendation at the regional scale, daily profiles of the DMS seawater concentrations from satellite data may improve modeling results.

Most of the regional studies using the CMAQ model employed the parameterization of LM86 to obtain DMS flux. However, the WRF-Chem model has been mostly applied using the parameterization of N00. Regarding the studies that have used the GEOS-Chem and other global models, most of them have also used the parameterization of LM86; however, several studies with the parameterization of N00 have also made significant contributions.

Although the differences shown in studies that have used both parameterizations are significant, future studies should apply those parameterizations with more scientific support for monitoring. The emissions using N00 can be up to double those obtained using LM86, which can impact on the results of DMS dispersion and its by-products. In other words, given the uncertainty of which parameterization to use, it is suggested to use the one that has been used in the study area and that has yielded results consistent with the study period.

## Figures and Tables

**Table 1.** Parameterizations to obtain the factor $k_{w}$.

Parameterization and Reference	Type	Equation
LM86 [26]	Linear	$k_{w}=0.17\cdot U_{10}\cdot {\left(\frac{Sc_{N}}{660}\right)}^{-0.5};U_{10}\left(\frac{m}{s}\right)\leq 3.6$
		$k_{w}=\left(2.85\cdot U_{10}-9.65\right)\cdot {\left(\frac{Sc_{N}}{660}\right)}^{-0.5};3.6<U_{10}\left(\frac{m}{s}\right)<13$
		$k_{w}=5.9\cdot U_{10}\cdot {\left(\frac{Sc_{N}}{660}\right)}^{-0.5};U_{10}\left(\frac{m}{s}\right)\geq 13$
W92 [27]	Quadratic	$k_{w}=0.31\cdot U_{10}^{2}\cdot {\left(\frac{Sc_{N}}{660}\right)}^{-0.5}$
WM99 [28]	Cubic	$k_{w}=0.0283\cdot U_{10}^{3}\cdot {\left(\frac{Sc_{N}}{660}\right)}^{-0.5}$
N00 [29]	Quadratic	$k_{w}=\left(0.222\cdot U_{10}^{2}+0.333\cdot U_{10}\right)\cdot {\left(\frac{Sc_{N}}{600}\right)}^{-0.5}$
H06 [30]	Quadratic	$k_{w}=0.266\cdot U_{10}^{2}\cdot {\left(\frac{Sc_{N}}{660}\right)}^{-0.5}$
M09 [31]	Linear	$k_{w}=\left(1.92\cdot U_{10}-1.0\right)\cdot {\left(\frac{Sc_{N}}{720}\right)}^{-0.5}$
W14 [32]	Quadratic	$k_{w}=0.251\cdot U_{10}^{2}\cdot {\left(\frac{Sc_{N}}{660}\right)}^{-0.5}$

**Table 2.** Regional modeling studies including DMS emissions and chemistry.

Model	Source of the DMS Emission Inventory	DMS Chemistry	Main Findings	Reference
CMAQ (v4.6)	Constant value from [55].	Reaction with radicals OH and ${\text{NO}}_{3}$ and halogen Cl.	The aerosol sulfate increased by nearly 2 μg·m^3^ in ocean and 0.1–0.2 μg·m^3^ in inland areas, attributable to DMS emissions and its chemistry.	[56]
CMAQ (v5.1)	DMS seawater concentration from [19] using the parameterization of LM86 [26].	Reaction with halogen compounds BrO and IO.	An increase of 10% in secondary organic aerosol mean concentration due to aerosol acidity and sulfate aerosol formation. Otherwise, the ozone and nitrogen dioxide $\left({\text{NO}}_{2}\right)$ concentrations diminished up to 5 ppbv and 2.5 ppbv.	[9]
CMAQ (v5.2)	DMS seawater concentration from [19] and local Chinese database using the parameterization of LM86, N00, W92 [26,27,29].	Reaction with radicals OH and ${\text{NO}}_{3}$ and halogen Cl. Also, the reactions with halogen compounds IO, BrO, and ClO were considered.	The inclusion of marine emissions of DMS and halogens increased by 4% the formation of sulfate aerosols on the coast and by 9% at sea.	[64]
CMAQ (v5.2)	DMS seawater concentration from [19] and local Chinese database using the parameterization of LM86 [26].	Reaction with radicals OH and ${\text{NO}}_{3}$ and halogen Cl. Also, the reactions with halogen compounds IO, BrO, and ClO were considered.	The DMS emissions increased ${\text{SO}}_{2}$ concentrations with the highest contribution in summer (0.63 μg/m^3^) and lowest in winter (0.12 μg/m^3^). The contribution of DMS to the ${\text{SO}}_{2}$ levels in Shanghai was mostly around 0.08 μg/m^3^. The inclusion of DMS reduced ${\text{O}}_{3}$ relatively larger in the open sea than in the urban area of Shanghai.	[10]
CMAQ (v5.3)	DMS seawater concentration from [19] using the parameterization of LM86 [26].	Reaction with radicals OH and ${\text{NO}}_{3}$ and halogen Cl. Also, the reactions with halogen compounds IO, BrO, and ClO were considered.	Total of 63.5% of DMS was oxidized by OH (33.0% via the abstraction channel and 30.5% via the addition channel). Also, the oxidation of DMS by ${\text{NO}}_{3}$ accounted for 11.8%. Therefore, BrO, Cl, IO, and ClO oxidation pathways contributed 16.0%, 8.2%, 0.4%, and 0.1%, respectively, to the total DMS oxidation.	[16]
CMAQ (v5.3.2)	DMS concentration was estimated using a DMS empirical algorithm constructed using chlorophyll a (Chl-a) concentrations, mixing layer depth, and using the parameterization of LM86 [26].	Reaction with radicals OH and ${\text{NO}}_{3}$ and halogen Cl. Also, the reactions with halogen compounds IO, BrO, and ClO were considered.	DMS contribution to the total PM_2.5_ mass was approximately 1%, showing more influence in the coastal area than the open ocean.	[65]
CMAQ (v5.3.2)	DMS concentration was estimated using a DMS empirical algorithm constructed using chlorophyll a (Chl-a) concentrations, mixing layer depth, and using the parameterization of LM86 [26].	Reaction with radicals OH and ${\text{NO}}_{3}$ and halogen Cl. Also, the reactions with halogen compounds IO, BrO, and ClO were considered.	Significant contributions of DMS emissions to PM_2.5_ concentrations in coastal areas. In addition, the mean contributions of DMS oxidation to ${\text{SO}}_{2}$ concentrations over marine and coastal areas were approximately 14.5% and 13.1%, respectively	[66]
CMAQ (v5.4)	DMS seawater concentration from [19] using the parameterization of LM86 [26].	Reaction with radicals OH and ${\text{NO}}_{3}$ and halogen Cl. The reactions of DMS with BrO and IO were not included in this study.	DMS emissions increased annual mean sulfate by 0.055 μg/m^3^ over the land area of the modeling domain. The inclusion of DMS emissions increased sulfate concentrations by 36% over seawater and 9% over land.	[67]
WRF-Chem	Not reported.	Chemical reactions reported in [72].	Added the coral-reef-derived DMS contribution, showing insignificant change (around 1%) to the total sulfate aerosol mass.	[73]
WRF-Chem	Constant value of DMS concentration in the seawater from VOCALS-Rex campaign.	Reaction with radicals OH and ${\text{NO}}_{3}$, without considering the formation of MSA.	The results were consistent with the surface measurement at the shipboard and showed good agreement with aircraft data.	[53]
WRF-Chem	Constant value of DMS concentration in the seawater using the parameterization of LM86 [26].	Chemical reactions reported in [72].	The study overestimated the DMS emissions due to overestimating the modeled DMS in the ocean due to the atmosphere transfer velocity, generating high biases in the modeled marine boundary layer and increasing the cloud-driven ${\text{SO}}_{2}$-to-sulfate conversion.	[61]
WRF-Chem	Constant value of DMS concentration in the seawater using the parameterization of N00 [29].	Reaction with radicals OH and ${\text{NO}}_{3}$, without considering the formation of MSA.	An overestimation of DMS flux was obtained but showed its impact in the albedo. Also, the simulation tests suggested that DMS influenced the aerosol number and size distribution.	[62]
WRF-Chem	Constant value without details about the parameterization applied.	Reaction with radicals OH and ${\text{NO}}_{3}$ and halogen Cl. Also, the reaction with halogen compound BrO was considered.	A nighttime ${\text{NO}}_{3}$ oxidation of DMS showed its relevance compared to daytime OH reaction. This particular finding probed the biases found before regarding DMS chemistry.	[63]
WRF-Chem	DMS seawater concentration from [19,40] using the parameterization of LM86, N00 [26,29].	Reaction with radicals OH and ${\text{NO}}_{3}$.	This study updated the climatology and parameterization in the configuration of the WRF-Chem model. The parameterization of N00 showed higher (double) MSA surface concentrations. Meanwhile, large uncertainties were present due to the avoided emissions of BrO from volcanic plumes.	[71]
WRF-Chem	Constant value of DMS concentration in the sea using the parameterization of N00 [29].	Not reported.	Did not analyze the effect of marine emissions.	[70]
WRF-Chem	Constant value of DMS concentration in the sea using the parameterization of N00 [29].	Reaction with radicals OH and ${\text{NO}}_{3}$.	A high source contribution (90–100%) of DMS to surface ${\text{SO}}_{2}$ in the Artic was reported. Meanwhile, the inclusion of DMS emissions increased 2–4% the concentrations of PM_10_. Adding DMS slightly reduced (−2 ppbv) the surface ozone over the ocean, even when several improvements in the performance of the model for ${\text{O}}_{3}$ and surface sulfate were applied. The simple gas phase chemistry had an impact on overestimation in some cases.	[69]
WRF-Chem	GOCART database	Reaction with radicals OH and ${\text{NO}}_{3}$.	Dust and particulate matter were analyzed, revealing an overestimation of PM_2.5_ when the dust module was considered, but neither discussion was done about the effect of marine emissions.	[56]
WRF-Chem	GOCART database	Reaction with radicals OH and ${\text{NO}}_{3}$.	Did not analyze the effect of marine emissions.	[57]
WRF-Chem	GOCART database	Reaction with radicals OH and ${\text{NO}}_{3}$.	Did not analyze the effect of marine emissions.	[58]
WRF-Chem	GOCART database	Reaction with radicals OH and ${\text{NO}}_{3}$.	Did not analyze the effect of marine emissions.	[59]
WRF-Chem	GOCART database	Reaction with radicals OH and ${\text{NO}}_{3}$.	Did not analyze the effect of marine emissions.	[60]
WRF-Chem	DMS seawater concentration from [19] using the parameterization of LM86 [26].	Chemical reactions reported in [72].	First research studying the role of coral-reef-derived DMS at sub-daily timescales. The authors used the version designed with 30 DMS oxidation pathway reactions. The inclusion of coral-reef-derived DMS resulted in no significant change in sulfate aerosol mass or total aerosol number. DMS removal by BrO or ${\text{Cl}}_{2}$ was not considered.	[68]
Enviro-HIRLAM	Used the parameterization of N00 [29] without details about the climatology used.	Reaction with radicals OH and ${\text{NO}}_{3}$.	An underestimation for PM_2.5_ was obtained, especially in coastal cities like Bilbao in Spain. Meanwhile, this study did not analyze the effect of DMS.	[74]
EMEP4UK-WRF	Monthly emission fields of DMS-derived ${\text{SO}}_{2}$ were taken from the work of [75].	Not reported.	Did not analyze the effect of DMS.	[76]
GEM-MACH	DMS seawater concentration from [19] and a satellite DMS concentration dataset from [47] using the parameterization of LM86 [26].	Reaction with radicals OH and ${\text{NO}}_{3}$.	The addition of DMS compartment to the GEM-MACH model resulted in a significant increase in atmospheric ${\text{SO}}_{2}$ for some regions of the Canadian Arctic (up to 100%). This study did not consider the impacts of halogens in the DMS chemistry. The climatology data of Lana et al. (2011) [19] do not well reflect the marine source in the Arctic due to the very limited observations available.	[77]
GEM-MACH	DMS seawater concentration from [19] and a satellite DMS concentration dataset from [47] using the parameterization of LM86 [26].	Reaction with radicals OH and ${\text{NO}}_{3}$.	Dimethyl sulfide oxidation led to increased aerosols between 60 and 200 nm and a 50% increase in droplet number in some regions of the Arctic.	[78]

**Table 3.** Global modeling studies including DMS emissions and chemistry.

Model	Source of the DMS Emission Inventory	DMS Chemistry	Main Findings	Reference
GEOS-Chem	DMS seawater concentration from [19,40,82] using the parameterization of N00 [29].	Reaction with radicals OH and ${\text{NO}}_{3}$ and the halogen compound BrO.	A small contribution (11–30%) of sea ice extent to DMS emissions, but this was responsible for 26–62% of MSA deposition at the Antarctic coast and 36–95% inland. Also, the lifetime of DMS and MSA was varied if its reaction with BrO was considered or not.	[83]
GEOS-Chem-REAM	Not reported.	Reaction with radical OH and the halogen compound BrO.	By adding 1 pptv BrO, a reduction of 13% to DMS to ${\text{SO}}_{2}$ conversion efficiency was obtained.	[84]
GEOS-Chem-TOMAS	DMS seawater concentration from [40] using the parameterization of LM86 [26].	Reaction with radicals OH and ${\text{NO}}_{3}$.	The results showed overprediction of MSA concentrations compared to observed registries at Alert station argued by overestimation of DMS emissions. Other explanations could be associated with missing reactions in the chemical mechanism that could sink DMS in that region. The results showed the main contribution to nuclei cloud droplets (CCNs) from DMS during July–August in the region.	[85]
GEOS-Chem	DMS seawater concentration from [19] improved with the 21-day ship track measurements, using the parameterization of LM86 [26].	The study applied DMS oxidation only by reaction with OH and ${\text{NO}}_{3}$ according to Equations (1)–(3) following [85].	The non-marine sources could contribute additional episodes of DMS in the Arctic region, although local marine sources of DMS dominated. Authors observed higher emissions during the monitoring campaign compared to the data reported in [19].	[86]
GEOS-Chem	DMS seawater concentration from [19] improved with the 21-day ship track measurements using the parameterization of LM86 [26].	Reaction with radicals OH and ${\text{NO}}_{3}$.	First attempt at comparison between aircraft measurements and simulated DMS at different levels of altitude. The model overpredicted the measurements at lower altitudes (below 1500 m).	[87]
GEOS-Chem	DMS seawater concentration from [19,40] without details about the parameterization applied.	Reaction with radicals OH and ${\text{NO}}_{3}$, with the halogen compound BrO, with the halogen Cl, and with ${\text{O}}_{3}$ (in multiphase).	The main results suggested a global oxidation of DMS in the gas phase by OH (66%), ${\text{NO}}_{3}$ (16%), and BrO (12%). This paper denoted the presence (around 86%) of DMS in tropospheric burden below 2 km. The reaction with BrO was considerable in the Southern Ocean and Antarctica during winter.	[17]
GEOS-Chem-TOMAS	DMS seawater concentration from [19] without details about the parameterization applied.	Reaction with radicals OH and ${\text{NO}}_{3}$.	The authors focused on the effect of MSA on the direct and indirect radiative force by the aerosols formed in 2014, obtaining negligible results (<−0.1 W m^−2^). However, uncertainties in DMS emission inventory and MSA transformation highlight the need for future studies to improve understanding in this area.	[88]
GEOS-Chem	The authors did not expose details about the DMS emission inventory.	The authors did not expose the DMS chemistry included in the model.	The results indicated low apportionment of DMS to particulate matter.	[89]
GEOS-Chem-TOMAS	DMS seawater concentration from [19] and DMS concentrations predicted by XGBoost using the parameterization of N00 [29].	The authors did not expose the DMS chemistry included in the model.	DMS contributed around 88% to sulfate in remote oceanic areas. Authors used machine learning techniques to improve the DMS concentrations in seawater for the emission inventory. Meanwhile, the authors suggested more marine and atmospheric observational data for further model evaluation.	[90]
HadGEM2-AO	DMS seawater concentration from [41] without details about the parameterization applied.	The authors did not expose the DMS chemistry included in the model.	DMS flux confirmed effects regarding temperature, cloud fraction, and radiation, especially in polar regions. Authors emphasized the urgent need for more studies about the synergy of the phytoplankton activity and the climate responses due to DMS flux.	[91]
HadGEM3-GA7.1	DMS seawater concentration from [19] using the parameterization of LM86 [26].	The authors did not expose the DMS chemistry included in the model.	The model responses were analyzed with combinations of seawater concentrations of DMS and parameterizations for sea–air gas transfer. Also, the authors used a scenario where the emission of DMS was multiplicated by the factor 1.7. The analysis was restricted to the region between 50° S and 70° S, considering the strong seasonal cycle of DMS emissions.	[92]
HadGEM3-GA7.1	DMS seawater concentration from [19] using the parameterization of LM86 [26].	Reaction with radicals OH and ${\text{NO}}_{3}$, with the halogen compound BrO, with the halogen Cl, and with ${\text{O}}_{3}$ (in multiphase).	The main finding of the study was the inclusion of halogen in the DMS chemistry. As a result, the updated pathway led to a 20% increase in the number concentration of cloud condensation nuclei and cloud droplets, showing better performance with observations, especially in the Southern Ocean between 1989 and 2008.	[93]
UKESM1, BCC-ESM1, GFDL-CM4, HadGEM3-GC3.1-LL, MRI-ESM2-0, NorESM2, GFDL-ESM4, MPI-ESM-1-2-HAM, MIROC-ES2L	No details about the climatology data applied, but the authors informed the use of the parameterization of LM86, W92, W14, N00 [26,27,29,32].	Not reported.	This paper focused on the wind speed changes impact on the DMS flux. Increased wind speeds over the summertime Southern Ocean resulted in increases of 0.82% in atmospheric DMS on average.	[94]
MIROC	Not reported.	Not reported.	Did not analyze the effect of marine emissions.	[95]
MIROC-ESM2	Not reported.	Not reported.	Did not analyze the effect of marine emissions.	[96]
CAM-Chem (version5)	AeroCom dataset	Not reported.	Did not analyze the effect of marine emissions.	[97]
NorESM2	DMS seawater concentration from [19] with the parameterization of W14 [32]. The flux of DMS was considered only unidirectional from the sea to the air.	Not reported.	Did not analyze the effect of marine emissions.	[98]
CESM	Used the method of [99] without details about the seawater concentration applied.	Only the DMS oxidation pathway with OH was used.	Showed the influence of El Niño–Southern Oscillation (ENSO) cycle on the DMS removal process in the air. Also, the authors found that DMS lifetime was about 3 days, and the ${\text{SO}}_{2}$ formation was 52% from the DMS oxidation, mainly increasing the cloud condensation nuclei formation over the eastern Pacific Ocean.	[100]
CESM-CAM5	Not reported.	Not reported.	This work found the source receptor apportionment of sulfate from 16 regions, being DMS from Southern Hemisphere responsible for 17–84% of the seasonal sulfate. DMS found to account for 16% of global mean sulfate concentrations.	[101]
CESM-CAM4	Not reported.	Reaction with radicals OH and ${\text{NO}}_{3}$, with the halogen compounds BrO, with the halogen Cl.	The addition of MeSH emissions showed competition with DMS for oxidants.	[12]
CanAM4.1	DMS seawater concentration from [19,40,41] using the parameterizations of LM86 and N00 [26,29].	Reaction with radicals OH and ${\text{NO}}_{3}$.	The global mean radiative effect of sulfate found to be approximately linearly proportional to the global mean surface flux of DMS. Included DMS emissions from the terrestrial biosphere, but they were not analyzed for their source effect. Meanwhile, more than 40% of sulfur originating from DMS emissions found to be responsible of the sulfate concentrations in the open ocean.	[102]
CanAM4.3	DMS seawater concentration from [19] using the parameterization of N00 [29].	Reaction with radicals OH and ${\text{NO}}_{3}$.	The model reproduced the seasonal variations, but an underestimation of DMS emissions was distinguished due to the omission of the sea ice melting source. Also, the authors found large uncertainties for nucleation parameterizations. The simulation overestimated the DMS source contribution to sulfate aerosol concentration at the Alert station.	[103]
ECHAM-HAM	DMS seawater concentration from [55] without details about the parameterization applied.	Not reported.	The results showed no effect of marine emissions during the simulation for one year and focused on anthropogenic emissions.	[104]
ECHAM-HAMMOZ	Not reported.	Reaction with radicals OH and ${\text{NO}}_{3}$, with the halogen Cl.	The sulfate offset found to be higher when the MSA formation potential due to the reactive uptake was lower, that is, higher DMS-to-${\text{SO}}_{2}$ oxidation.	[105]
STOCHEM-CRI	DMS seawater concentration from [19] without details about the parameterization applied.	Reaction with radicals OH and ${\text{NO}}_{3}$, with the halogen compound BrO, with the halogen Cl.	One of the main findings was 7.9% of the DMS reacted with BrO. BrO oxidation contributed significantly in the high latitudes of the Southern Hemisphere. A large amount of DMS was removed via reaction with Cl, specifically in the remote Southern Hemisphere ocean.	[106]

## Data Availability

No new data were created or analyzed in this study.

## Abbreviations

The following abbreviations are used in this manuscript:

3GP: Three main gas phase
AerChemMIP: Aerosols and 47 Chemistry Model Intercomparison Project
CAM-Chem: The Community Atmosphere Model with Chemistry
CAMS: Copernicus Atmosphere Monitoring Service
CanAM: The Canadian Atmospheric Model
CCN: Cloud condensation nuclei
${\text{CH}}_{3}{\text{O}}_{2}$: Methyl peroxy radical
Chl-a: Chlorophyll a
CMAQ: Community Multiscale Air Quality
CMIP6: Coupled Model Intercomparison Project
${\text{C}}_{\text{g}}$: Concentration of DMS in air
${\text{C}}_{\text{w}}$: Concentration of DMS in water
DMS: Dimethyl sulfide
DMSO: Dimethyl sulfoxide
DMSP: Dimethysulfoniopropionate
ECCAD: Emissions of Atmospheric Compounds and Compilation
ECHAM: European Centre Hamburg general circulation model
EMD: DMS equivalent mixing depth
EMEP4UK: European Monitoring and Evaluation Programme for United Kingdom
Enviro-HIRLAM: Environment—High Resolution Limited Area Model
F: Sea–air flux
GEM-MACH: Global Environmental Multiscale—Modelling Air quality and Chemistry
GEOS-Chem: Goddard Earth Observing System—Chemistry
GOCART: Goddard Chemistry Aerosol Radiation and Transport
H: Henry’s law constant
HadGEM: Hadley Centre Global Environmental Model
H06: Parameterization from Ho et al., (2006) [30]
${\text{HNO}}_{3}$: Nitric acid
IOMB: International Ocean Model Benchmarking
IPCC: Intergovernmental Panel on Climate Change
IRR: Integrated Reaction Rate
${\text{k}}_{\text{a}}$: Total resistance to gas transfer on side of the air
${\text{k}}_{\text{w}}$: Total resistance to gas transfer on side of the sea
LM86: Parameterization from Liss and Merlivat (1986) [26]
M09: Parameterization from Marandino et al., (2009) [31]
MIROC: Model for Interdisciplinary Research on Climate
MSA: Methanesulfonic acid
N00: Parameterization from Nightingale et al., (2000) [29]
${\text{NO}}_{3}$: Nitrate radical
NorESM2: The Norwegian Earth System Model version 2
OH: Hydroxyl radical
PASE: Pacific Atmospheric Sulfur Experiment
R: Universal gas constant
REAM: The Regional chEmical trAnsport Model
${\text{S}}_{\text{cn}}$: Normalized Schmidt number
${\text{SO}}_{2}$: Sulfur dioxide
VSL: Very short-lived
T: Seawater temperature
${\text{T}}_{\text{a}}$: Temperature of the air
TOMAS: TwO-Moment Aerosol Sectional
${\text{U}}_{10}$: Wind speed at 10 m
VOCALS-Rex: VAMOS Ocean-Cloud-Atmosphere-Land Study Regional Experiment
W14: Parameterization from Wanninkhof et al., (2014) [32]
W92: Parameterization from Wanninkhof (1992) [27]
WM99: Parameterization from Wanninkhof et al., (1999) [28]
WRF-Chem: Weather Research and Forecasting coupled with Chemistry
